# Novel neuroprotective therapy with NeuroHeal by autophagy induction for damaged neonatal motoneurons

**DOI:** 10.7150/thno.43765

**Published:** 2020-04-06

**Authors:** David Romeo-Guitart, César Marcos-DeJuana, Sara Marmolejo-Martínez-Artesero, Xavier Navarro, Caty Casas

**Affiliations:** Institut de Neurociències (INc) and Department of Cell Biology, Physiology and Immunology, Universitat Autònoma de Barcelona (UAB), & Centro de Investigación Biomédica en Red sobre Enfermedades Neurodegenerativas (CIBERNED), Bellaterra, Barcelona, Spain.

**Keywords:** neonatal motoneurons, neuroprotection, autophagy, SIRT1/AKT/FOXO3a, NeuroHeal

## Abstract

**Rationale**: Protective mechanisms allow healthy neurons to cope with diverse stresses. Excessive damage as well as aging can lead to defective functioning of these mechanisms. We recently designed NeuroHeal using artificial intelligence with the goal of bolstering endogenous neuroprotective mechanisms. Understanding the key nodes involved in neuroprotection will allow us to identify even more effective strategies for treatment of neurodegenerative diseases.

**Methods**: We used a model of peripheral nerve axotomy in rat pups, that induces retrograde apoptotic death of motoneurons. Nourishing mothers received treatment with vehicle, NeuroHeal or NeuroHeal plus nicotinamide, an inhibitor of sirtuins, and analysis of the pups were performed by immunohistochemistry, electron microscopy, and immunoblotting. *In vitro*, the post-translational status of proteins of interest was detailed using organotypic spinal cord cultures and genetic modifications in cell lines to unravel the neuroprotective mechanisms involved.

**Results**: We found that the concomitant activation of the NAD^+^-dependent deacetylase SIRT1 and the PI3K/AKT signaling pathway converge to increase the presence of deacetylated and phosphorylated FOXO3a, a transcription factor, in the nucleus. This favors the activation of autophagy, a pro-survival process, and prevents pro-apoptotic PARP1/2 cleavage.

**Major conclusion**: NeuroHeal is a neuroprotective agent for neonatal motoneurons that fine-tunes autophagy on by converging SIRT1/AKT/FOXO3a axis. NeuroHeal is a combo of repurposed drugs that allow its readiness for prospective pediatric use.

## Introduction

Neurons, like other cells, responds to stress by engaging both pro-survival subprograms, which we collectively term endogenous mechanisms of neuroprotection, and cell death subprograms. Depending on the intensity of injury, the balance between these subprograms results in either neuronal survival or death 1. These mechanisms of neuroprotection include the unfolded protein response (UPR), the heat-shock response, the autophagy pathway, the ubiquitin-proteasome system, chaperone systems, the endoplasmic reticulum-associated degradation machinery, and the antioxidant defense. Excess damage as well as aging result in defective functioning of one or more of these subprograms. We reasoned that favor their function in pathological conditions may be a successful strategy for neuroprotection. With this idea in mind, we discovered NeuroHeal, a new neuroprotective agent for adult axotomized spinal motoneurons (MNs), using a top-down strategy based on a network-centric approach, the use of artificial intelligence, and proper preclinical animal models 2. For this discovery, we took advantage of the successful engagement of endogenous mechanisms by MNs that allow their survival after distal axotomy (e.g., following distal nerve transection and suture of the sciatic nerve) 3-5, in contrast to the neurodegenerative process engaged after proximal axotomy (e.g., after nerve root avulsion) 6-8.

Nerve injuries, such as facial palsy or brachial plexus avulsion during obstetric interventions, provoke axonotmesis or neurotmesis depending, for instance, whether the injury is preganglionic or postganglionic. Motor function recovery after preganglionic avulsion injuries does not occur spontaneously, and hence surgical reconstruction is needed. Functional recovery is incomplete so that substantial chronic impairment will affect the whole life of the infant 9. The proper early timing of the surgical intervention for functional recovery in children is crucial. The critical dependency of the developing motoneurons (MNs) on their interaction with their target muscle 3,4 makes them quickly succumb after axotomy. Without survived MNs, surgical reimplantation of nerves is averted and justify the urgent need for pediatric neuroprotective agents.

Hence, we sought to test whether NeuroHeal is capable to rescue target-deprived neonatal MNs in a model of nerve injury in newborn rats as well as to identify key nodes for neonatal neuroprotection. In this model, in contrast to what happens to adult MNs, distal axotomy performed during the first two weeks of life lead to massive MN death 10,11. The death of axotomized neonatal motoneurons is linked to excitotoxicity 12 and occurs by apoptosis 13-15 In contrast, adult ventral horn spinal MNs presented abnormal response to endoplasmic reticulum stress and autophagy flux blockage as key events in their non-apoptotic neurodegenerative process 6-8. We found that the neuroprotective effect of NeuroHeal for adult MNs depends on the activation of the NAD^+^-dependent deacetylase sirtuin 1 (SIRT1) 2,16. SIRT1 has emerged as an important player in neurodegenerative diseases, after stroke, and in peripheral and cranial nerve injuries 17 and is tightly linked with cellular metabolic status and longevity. SIRT1 has a widely range of targets in both the cytosol and the nucleus, and modulates different functions such as inflammation, apoptosis, and autophagy 18. Thus, we started to focus on this molecule as one of the key nodes in the action of NeuroHeal, as well as other possible pro-survival nodes such as the phosphatidylinositol-4,5-bisphosphate 3-kinase(PI3K) /AKT axis, which suppresses apoptosis 19 although it may inhibit autophagy 20. We aimed to test whether NeuroHeal treatment may exert neuroprotection to axotomized neonatal MNs and to unravel the mechanism involved, which possibly implies the interplay between SIRT1 and PI3K/AKT.

## Materials and Methods

### Surgical procedures

All the procedures involving animals were approved by the Ethics Committee of the *Universitat Autònoma de Barcelona* and *Generalitat de Catalunya* and followed the European Community Council Directive 2010/63/EU. Pregnant rats were maintained under standard conditions of light and temperature and fed *ad libitum*. Once born, the litter was individualized for each rat and maintained with under the same conditions. For surgical intervention, we deeply anesthetized 4-day-old Sprague Dawley rat pups by hypothermia and dissected out the right sciatic nerve. The nerve was crushed twice with a fine forceps (Dumont #5) for 30 s. We closed the wound with sutures, and animals were allowed to recover in a warm environment and returned to the mothers.

### Drug treatments

NeuroHeal is a combination of acamprosate and ribavirin 2. For treatment with NeuroHeal, we ground acamprosate (Merck) and ribavirin (Normon) into fine powders and added to drinking water at final concentrations of 2.2 mM and 1 mM, respectively. NeuroHeal and 5 mM nicotinamide (NAM) (Sigma-Aldrich) were added to drinking water given to the mothers on the day prior to injury to ensure that the pups began to receive drug on the day of the injury. Drug or vehicle (water) treatments were maintained by refreshing water every 3 days.

### Histology and immunohistochemistry

At 10 dpi, 4 animals per group the animals were euthanized with dolethal (60 mg/kg, intraperitoneal) and were perfused with a transcardial infusion of a saline solution containing heparin (10 U/mL) followed by 4% paraformaldehyde in 0.1 M PBS. We collected the L4-L5 spinal cord segments, post-fixed for 2 h with 4% paraformaldehyde in 0.1 M PBS and introduced into a 30% sucrose solution for cryopreservation at 4 ºC. Tissue-tek embedded spinal cord samples were serially cut (20-µm sections) in a cryotome (Leica) and were kept at -20 ºC until analysis. For immunolabelling, we washed samples with Tris-buffered saline (TBS), with 0.1 mM glycine in TBS, and with blocking solution (0.3% Triton-X-100 and 10% donkey serum in TBS) for 1 h at room temperature. Samples were then incubated overnight at 4 ºC with primary antibodies (see Table S1). After several washes with 0.1% Tween-20 in TBS, we added donkey-Cy3 or Alexa488 against the primary antibody (1:200; Jackson Immunoresearch) and incubated for 1 h and 30 min at room temperature. Counterstaining was performed with fluorescent green or blue NeuroTracer Nissl Stain (Molecular Probes) and/or DAPI (Sigma) and mounting with Fluoromount-G mounting medium (SouthernBiotech). Samples from groups to be compared were processed and analyzed together on the same slide. Confocal images were systematically acquired using a confocal Laser Scanning Microscope (Zeiss LSM 700) using three separate photomultiplier channels with a 1.4 numerical aperture objective of 20X under the same conditions of sensitivity, resolution, and exposure time for each analyzed marker. Images were separately projected and merged using a pseudocolor display. Signal intensity was analyzed using ImageJ software (National Institutes of Health; available at http://rsb.info.nih.gov/ij/). The Nissl or DAPI labelling were used to identify cytosol or nucleus, respectively, and the integrated densities within these regions of interest were obtained for at least 15 MNs extracted from three different sections (separated by at least 100 μm) per animal for each marker.

### *In vitro* cultures

For *in vitro* studies, acamprosate, ribavirin, Ex-527, NAM, 3MA, LY-294-002 hydrochloride, and Staurosporine (St) (Sigma-Aldrich) and Bafilomycin A1 (Santa Cruz Biotechnologies) were diluted into water, DMSO or EtOH according to manufacturer's instructions. Concentrations used in cell culture were 55 µM, 1 µM, 10 µM, 5 mM, 10 µM, 10 µM, and 50 nM respectively. NSC-34 cells were cultured in modified Eagle's medium high-glucose (DMEM, Biochrom) supplemented with 10% fetal bovine serum (Sigma- Aldrich), 100 U/mL penicillin (Sigma-Aldrich), and 0.5 X penicillin/streptomycin solution (Sigma- Aldrich) and maintained with a humidified incubator at 37 ºC under 5% CO_2_, essentially as described in a previous report 6. For nucleotransfection, we used the Amaxa cell line Nucleofector II Kit R (Lonza) and the Nucleofactor V Kit (Lonza) following the manufacturer's recommendations. After 24 h of drug treatment with St (10, 20, and 50 nM) with and without NeuroHeal, cells were incubated with 4 mg/mL 3-(4,5-dimethylthiazol-2-yl)-2,5-diphenyltetrazolium bromide (MTT) solution for 1 h, medium was removed, and the resulting formazan salts were dissolved in DMSO. Absorbance was measured with a microplate reader (Bio-tek, Elx800) at 570 nm, and the percentage of cell survival was calculated relative to the control on the same plate.

### Spinal organotipics cultures

For spinal cord organotypic cultures (SOCs), we removed the spinal cords from 7-day-old Sprague Dawley rats and placed them in cold 30% glucose Gey's Balanced Salt Solution (Sigma-Aldrich). We removed meninges and cut spinal cords into 350-μm thick slices, which were placed onto Millicell-CM of 30-mm diameter (0.4 μm, Millipore) within wells of 6-well plates (Thermo Fisher Scientific) containing 1 mL of culture medium (50% (v/v) minimal essential medium (MEM), 2 mM glutamine, 25 (v/v) Hank's Balanced Salt Solution (Sigma-Aldrich) supplemented with 25.6 mg/mL glucose and 25 mM HEPES, pH 7.2 as described previously 21,22. Cultures were maintained in a 5% CO_2_, humidified environment at 37 ºC. The following day, we changed the medium and added drug or vehicle: Neuro, NeuroHeal+Ex-527, NeuroHeal+NAM, NeuroHeal+3-MA, or NeuroHeal+LY294, water or DMSO. Medium was changed twice per week. After 2 weeks of treatment, we removed the medium, post-fixed the spinal cord slices with cold 4% paraformaldehyde (PFA) solution in 0.1 M PBS, pH 7.2, for 1 h, washed them with TBS several times, and incubated for 48 h with primary antibodies combined with mouse-anti SMI32 (1:1000; Biolegend) at 4 ºC. Confocal microphotographs of a predefined Z-stack were taken covering ventral horn of each SOC, and MN presence was assessed by counting SMI32-positive neurons for each SOC hemisection (n=8-12 slices/condition).

### Motor neuron counting

We stained 20 slides (separated by 100 µm) covering all the L4-L5 medullar segments of each animal (4 animals per group) with FluoroNissl green (Life Technologies) for 20 min following the manufacturer's protocol. We took sequential microphotographs from at least 20 slices of the lateral funiculus from contra- and ipsilateral sides of each animal with the aid of a digital camera (Olympus DP76) attached to a microscope (Olympus BX51) at 20X. Only those MNs localized in the more lateral neuron pool of the grey matter with a prominent nucleus and soma of at least 900 µm^2^ were counted as MNs. The percentage of surviving MNs was calculated as the number of surviving MNs at the ipsilateral side with respect to the contralateral side for each animal.

### Western blotting and immunoprecipitation

For immunoblotting, 4 pooled SOC sections for each condition after 3 DIV (n=4 different treatments), pup spinal cord L4-L6 segments at 3 dpi (n=4), or NSC34 cell extracts obtained at 6 h post-treatments (n=4) were added to lysis buffer (50 mM Tris, pH 6.8, 2 mM EDTA, 0.5% Triton-X-100, and a cocktail of proteases (Sigma-Aldrich) and phosphatase inhibitors (Roche)). Samples were homogenized with a Pellet pestle (Sigma-Aldrich) on ice, and sonicated with a Ultrasonic homogenizer (Model 3000, Biologics Inc.). Then they were centrifuged for 10 min at 13000 g at 4 ºC, and protein in supernatants were quantified by BCA assay (Pierce Chemical Co.). Proteins (10 µg/well) were resolved by SDS-PAGE and transferred to a PVDF membrane in a BioRad cuvette system in 25 mM Tris, pH 8.4, 192 mM glycine, and 20% (v/v) methanol. We blocked the membrane with 5% milk solution in 0.1% Tween-TBS (TBS-T) for 1 h at room temperature and incubated overnight with primary antibodies (see Table S2). After washing in TBS-T, the membrane was incubated with an appropriate secondary antibody conjugated with horseradish peroxidase (1:5000, Vector) for 1 h. The membrane was visualized using a chemiluminescent method (ECL Clarity Kit, Bio-Rad Laboratories), and the images were captured and quantified with Image Lab Software (Bio-Rad Laboratories).

For immunoprecipitation, we followed the manufacturer's protocol (Life Technologies). Briefly, we linked the anti-acetylated lysine antibodies (1:200; Sigma) to magnetic particles by incubation on a rotatory wheel for 10 min at room temperature, washed, and incubated with 20 µg of protein extract by incubation for 10 min at room temperature on a rotatory wheel. After elution, proteins were denatured, resolved by SDS-PAGE, transferred to a PVDF membrane, and incubated with primary and secondary antibody as described above.

### Real-time PCR

Pups at 3 dpi (n=4) were euthanized, and the lumbar segment L4-L5 was removed. Total RNA was extracted using the RNeasy Plus Mini Kit (QIAGEN) according to manufacturer's instructions. RNA concentrations and quality were measured with a NanoDrop 2000 (Thermo Fisher Scientific). Reverse transcription was carried out using iSCript cDNA Synthesis Kit (BioRad), and the reaction was performed on a SureCycler 8000 Thermal Cycler (Agilent). Finally, we performed PCR using cDNA diluted in sterile water as template for primers listed in supplemental Table S3 using the brilliant III Ultra-Fast SYBR Green QPCR Master Mix (Agilent Technologies). Samples were plated in duplicate in a 96-well PCR plate and were analyzed using the MyiQ Single Colour Real-Time PCR Detection System (BioRad) and the Bio iQ5 Optical System Software (BioRad).

### Transmission electron microscopy

Perfused spinal cords in a fixative solution of 2.5% (v/v) glutaraldehyde (EM grade, Merck) and 2% (w/v) PFA in 0.1 M PBS, pH 7.4 were incubated for 2 h at room temperature on a rocking platform. Samples were fixed in 1% (w/v) PFA and subsequently post-fixed with 1% (w/v) osmium tetroxide (TAAB Laboratories) containing 0.08% (w/v) potassium hexacyanoferrate (Sigma-Aldrich) in PBS, pH 7.4 for 2 h at 4 ºC. After four washes with deionized water, we dehydrated samples with sequential washes of acetone. We embedded samples in EPON resin and polymerized at 60 ºC for 48 h and then cut into semi- thin sections (1 µm) with a Leica ultracut UCT microtome (Leica Microsystems). Sections were stained with 1% (w/v) aqueous toluidine blue solution and examined with a light microscope to identify the ventral horn area. Ultra-thin sections (70 nm) were then cut with a diamond knife, placed on coated grids, and contrasted with conventional uranyl acetate and Reynolds lead citrate solutions. Finally, we observed the sections with a JEM-1400 transmission electron microscope (Jeol) equipped with a Gatan Ultrascan ES1000CCD camera. We analyzed three to four MNs per animal (n=4 per group).

### Statistical analysis

Data are presented as means ± standard error of the mean (SEM). Statistical analyses were conducted using GraphPad Prism 5 software. We performed an unpaired t-test to compare two groups and one-way analysis of variance (ANOVA) to compare three or more groups followed by Bonferroni's multiple comparison test. Differences were assumed to be significant for p < 0.05.

## Results

### SIRT1 activation is necessary for survival of neonatal axotomized MNs

**Figure 1:**
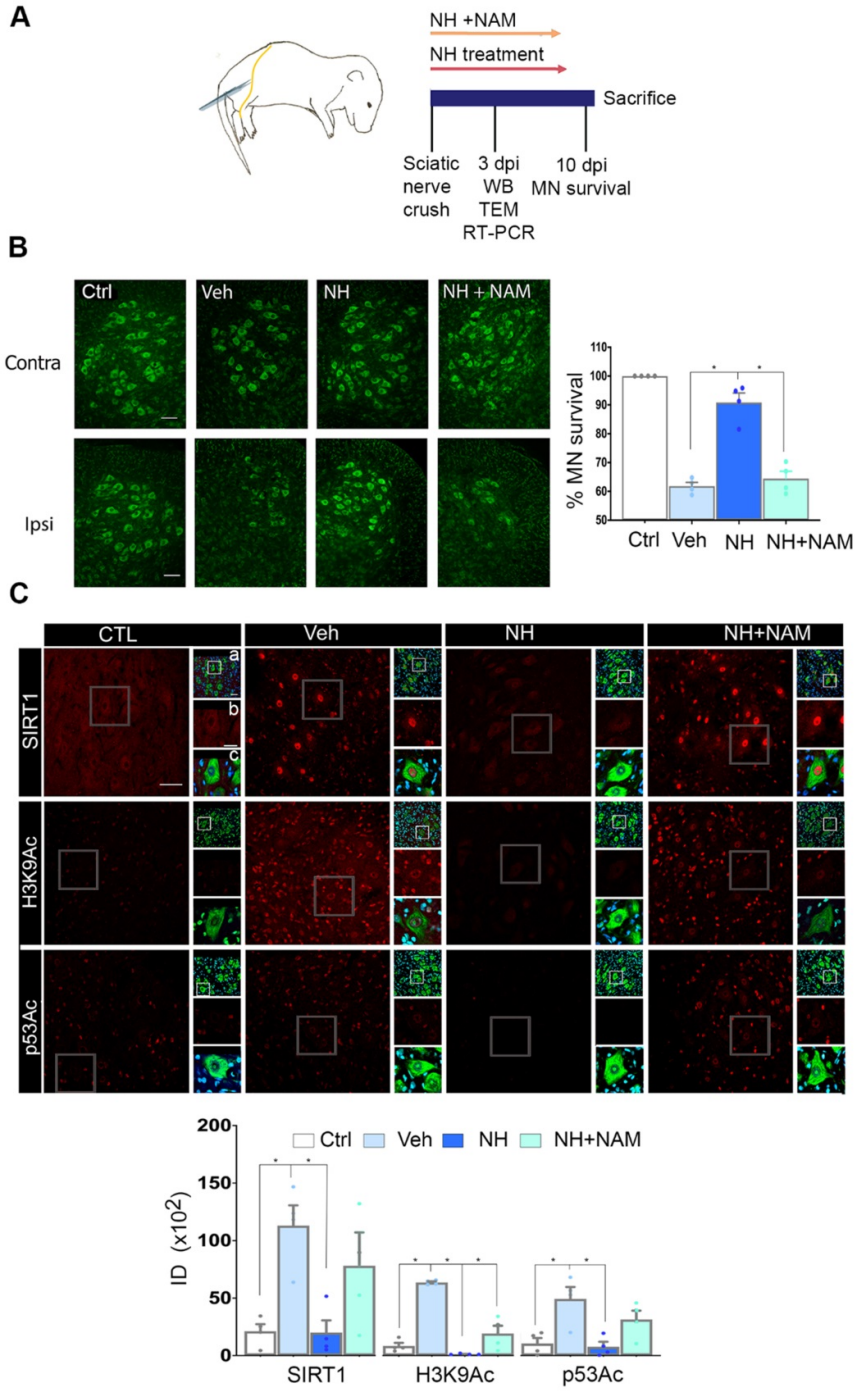
** SIRT1 activation mediates neuroprotection of neonatal axotomized motoneurons by NeuroHeal treatment. (A)** Schematic experimental workflow: sciatic nerve crush was performed on pups of 4 days old which were returned to mothers thereafter. Mothers were orally treated with either vehicle (Veh), NeuroHeal (NH), or NH + nicotinamide (NAM) for up to 3 or 10 days post injury (dpi) when pups were collected to perform transmission electron microscopy (TEM), Real-Time PCR (RT-PCR), western blot (WB), or immunohistochemical analyses, respectively. **(B) *Left*,** Representative microphotographs of FluoroNissl-stained (green) spinal cord sections from non-injured control (Ctrl) or injured vehicle-treated (Veh) animals at 10 dpi. Scale bar 100 μm at all images. ***Right***, Graph of the percentage of MNs found at the ipsilateral (crushed) with respect to the contralateral anterior spinal cord sides, from vehicle-treated (Veh), NH and NH+NAM groups (n=4 per group, ANOVA, post hoc Bonferroni *p<0.05). (**C**) ***Top***, Confocal images of MNs immunolabelled to reveal SIRT1 (red), and its downstream substrates: H3K9Ac (red), and p53Ac (red), and counterstained with FluoroNissl (green) and DAPI (blue), from the different animal groups at 10 dpi. At each condition, panels a-c are zoom images: a and c panels are merged microphotographs, and b panels are the amplified imaged of the corresponding single labeling in red. Scale bar 50 μm and identical for all corresponding microphotographs as represented in the first image panel, the Ctrl/SIRT1 condition. ***Bottom,*** Graph of the means of the immunofluorescence density (ID) for each marker inside the nuclei of MNs (n=4 per group, ANOVA, post hoc Bonferroni, *p<0.05).

We first established the neuroprotective effect of NeuroHeal treatment in a model of neonatal axotomy. It is known that neonatal axotomy of the sciatic nerve on or before postnatal day 4 triggers retrograde MN apoptosis 23-25. We treated mothers of the pups with either vehicle or NeuroHeal through drinking water until sacrifice of pups according to workflow planning (Figure 1A). Since one of the main NeuroHeal targets is SIRT1, a group of mothers was treated with NeuroHeal plus nicotinamide (NAM) as NAM is also an inhibitor of SIRT1 26. Axotomy resulted in death of 38% of L4-L5 ipsilateral anterior MNs, measured relative to the contralateral no- lesioned side at 10 days post injury (dpi), as expected (Figure 1B). NeuroHeal treatment significantly increased the number of axotomized MN survival, but NAM co- treatment blocked this neuroprotective effect (Figure 1B). We confirmed that SIRT1 was activated by NeuroHeal by analysis of SIRT1 substrates such as histone H3 acetylated at Lys9 (H3K9Ac) and p53 acetylated at Lys373 (p53Ac) 27,28. Using immunohistochemical analysis, we observed that axotomy resulted in SIRT1 nuclear accumulation and an accumulation of both H3K9Ac and p53Ac (Figure 1C). Treatment with NeuroHeal normalized the immunofluorescence intensity within MNs of these three proteins but in pups co-treated with NAM, the levels were similar to those in untreated animals (Figure 1C).

**Figure 2:**
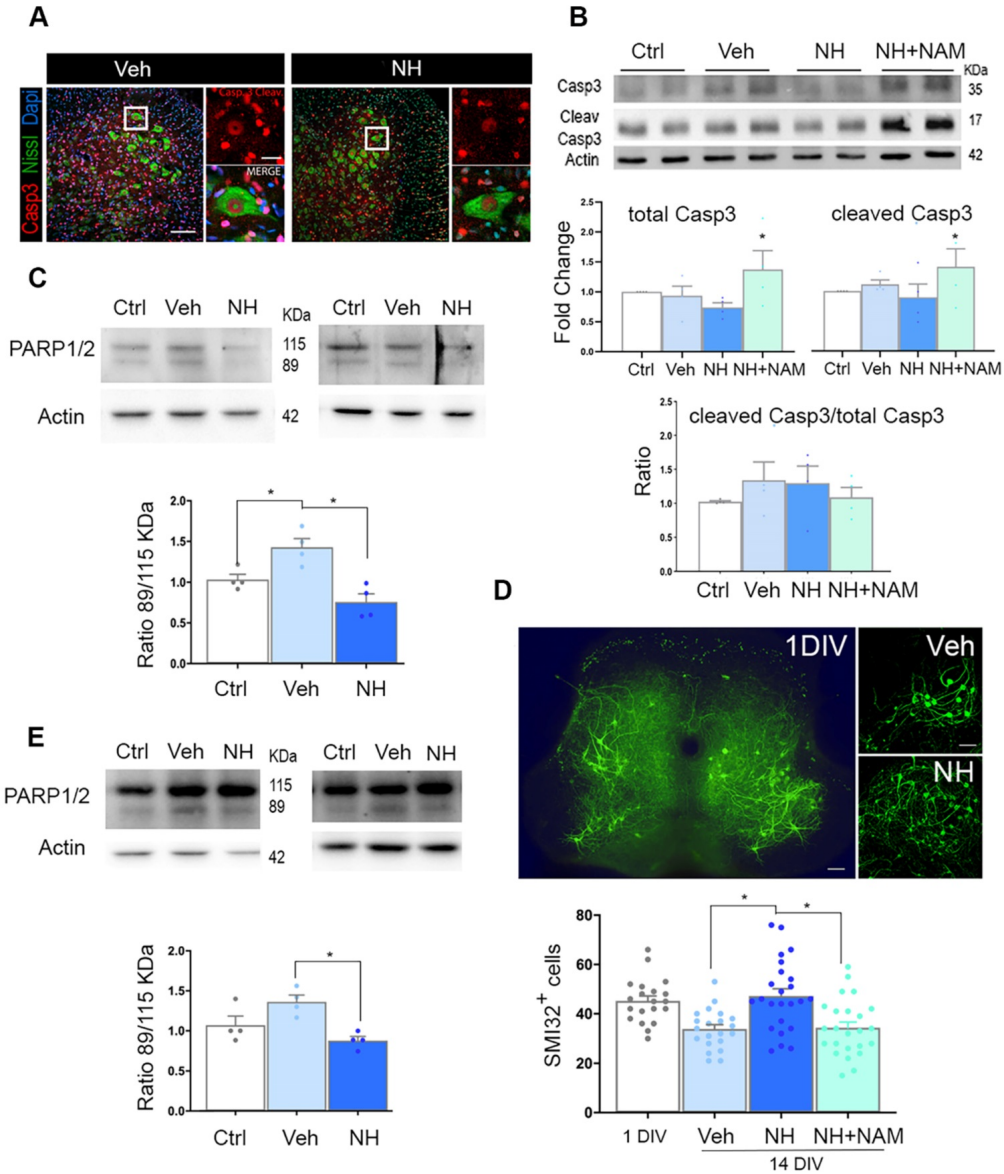
** NeuroHeal mediates neuroprotection from apoptosis at PARP1/2 level. (A) *Left,***Representative confocal images of neonatal axotomized MNs immunolabelled to reveal active cleaved caspase 3 (Casp3) (red), FluoroNissl (green), and DAPI (blue), from injured animals treated with vehicle (Veh) or NeuroHeal (NH) at 10 dpi. Scale bar 100 µm high magnification, and 20 µm low magnification. (**B**) Immunoblotting and corresponding graphs of the analysis of the ratio of cleaved Casp3 *vs* the total form in the different groups. (**C**) Representative western blot membrane and bar graph showing the protein fold change analysis of PARP1/2 levels in uninjured (Ctrl) and injured animals treated with and without NH at 3 dpi (n=4 per group, ANOVA, post hoc Bonferroni, *p<0.05). **(D) *Top,***Representative microphotographs of SMI-32-labelled MNs at the whole spinal cord slice (in 1day-in-vitro culture (DIV)) or at their ventral horns at 14 DIV from vehicle-, or NH-treated spinal cord organotypic cultures (SOCs). Scale bar 100 µm. ***Bottom,***Bar graphs showing the mean number (± SEM) of SMI-32-positive neurons in the ventral horn of each hemisection of the spinal cord slice (n=8-12 complete spinal cord slices, ANOVA, post hoc Bonferroni *p<0.05). (**E**) Western blot and associated bar graph showing the analysis of PARP1/2 protein levels from different treatment conditions of 14DIV SOCs (untreated Ctrl, or treated with vehicle or NH) (n=4 per group, ANOVA, post hoc Bonferroni, *p<0.05).

At 10 dpi, we observed copious staining of cleaved caspase 3 (Casp3) in nuclei of damaged MNs, and other cells, possible glial cells, at the ipsilateral ventral horn, as expected 25. The presence of cleaved Casp3 in glial cells has been reported by other authors previously in a model of intracortical neuronal excitotoxicity 29 or more recently due to exercise 30, claiming not necessarily link to apoptosis in these cells. Surprisingly, in axotomized MNs, we observed persistent cleaved Casp3 pattern in the group treated with NeuroHeal (Figure 2A). Accordingly, we observed similarly by immunoblotting, with no differences in the ratio between cleaved Casp3 versus total Casp3 among groups, including the group treated with NeuroHeal plus NAM despite higher levels of each of these forms (Figure 2B). We did not explore other casp3-independent apoptotic mechanisms since it was well established the predominant role of this caspase in the apoptotic neuronal demise by axotomy in pups 15. Hence, we hypothesized that NeuroHeal may modulate factors downstream of Casp3, such as PARP1/2, to exert the neuroprotective effect 31. Cleavage of PARP1/2 by caspases typically yields 89-kDa fragments that are considered hallmarks of apoptosis. In injured spinal cord L4-L5 extracts there was an increase in the amount of 89-kDa PARP1/2 fragment compared to levels in extracts of uninjured samples as expected (Figure 2C). NeuroHeal treatment reduced levels of full-length PARP1/2 (115-kDa band) and also the abundance of the 89-kDa form (Figure 2C). In agreement with this, immunohistochemical analysis of PARP1/2 localization revealed higher levels within the nuclei and the cytosol of axotomized MNs compared to uninjured control where slighter levels were observed in the nucleus (Figure S1). Other nuclei were also stained in surrounding cells, possibly glial cells. The raised of PARP1/2 after damage is expected as well as the nuclear and cytosolic presence. We have used a pan antibody, the same as for immunoblotting, that did not distinguish both forms by immunohistochemistry. However, full-length PARP1/2 is predominantly nuclear, but the 89kDa-fragment is liberated from the nucleus into the cytosol 31. NeuroHeal treatment drastically reduced overall PARP1/2 levels, hardly detected only in the nucleus of MNs and other surrounding cells, as in the control animals. In contrast, NAM co-treatment abolished the effect produced by NeuroHeal with MNs saturated of PARP1/2 in both nuclei and cytosol (Figure S1). This result suggested that NeuroHeal might prevent late apoptotic events somehow, and considering that PARP1/2 can be degraded by autophagy 32 we explored the existence of these process further down.

To confirm these results *in vitro*, we used a previously validated model of neonatal axotomy based on spinal organotypic culture (SOC) 21. In spinal cord slices devoid of roots, around 25% of MNs died by 14 days of *in vitro* culture (DIV) compared to the number of neurons present by 1 DIV (Figure 2D). We observed that treatment with NH during 14 days sustained neuronal population. This neuroprotective effect was abolished with the addition of NAM to the culture suggesting that SIRT1 activation is necessary. (Figure 2D; *p*<0.05). Accordingly, axotomy provoked accumulation of the 89-kDa PARP1/2 fragment, and this was attenuated by NeuroHeal treatment (Figure 2E;* p*<0.05). Hence, the *in vitro* model of axotomy reproduced the phenomena observed *in vivo*.

### NeuroHeal-induced neuroprotection sustain both SIRT1 and AKT activation and downstream FOXO3a modifications

**Figure 3:**
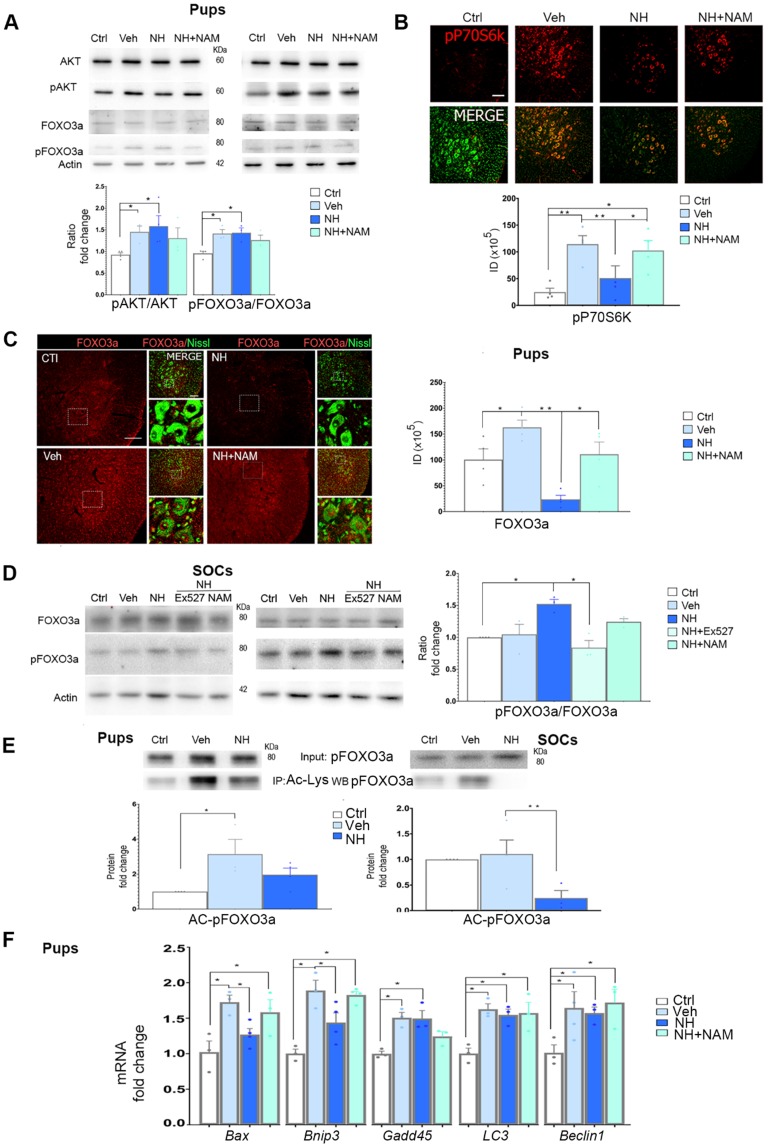
** NeuroHeal modulates FOXO3a activity.** (**A**) Immunoblots and bar graphs showing the analysis of pAKT related to total AKT levels, and pFOXO3a related to its total form in the different experimental groups (uninjured (Ctrl), vehicle (Veh), NeuroHeal (NH) or NH plus nicotinamide (NAM) treated animals) at 3 dpi. (n=4 animals, ANOVA, post hoc Bonferroni, *p<0.05). (**B**) Confocal microphotographs and graphs showing the immunofluorescent density of pP70S6k (red) co-labelled with FluoroNissl (green) from different experimental groups. Scale bar 100 µm. (n=4 animals, ANOVA, post hoc Bonferroni, *p<0.05, **p<0.01) (**C**) ***Left,***Microphotographs showing the localization and levels of total FOXO3a (red), co-labelled with FluoroNissl (green) in different experimental groups at 10 dpi. Scale bar 100 µm (FOXO3a) and 10 µm (in zoomed merged image). ***Right***, Bar graph showing the percentage of nuclear respect to whole cell immunofluorescence of FOXO3a within MNs at the ventral horns from the different experimental conditions (n=4 per group, ANOVA, post hoc Bonferroni, *p<0.05, **p<0.01). (**D**) Western blots and graphs showing the analysis of pFOXO3a respect to total FOXO3a in SOCs treated as indicated (n=4 per group, ANOVA, post hoc Bonferroni, *p<0.05). (**E**) Western blots and graphs showing the analysis of immunoprecipitated acetylated pFOXO3a (Ac-pFOXO3a) levels in different experimental groups at 3 dpi from injured pups (left) or SOCs (right) (n=4 per group, ANOVA, post hoc Bonferroni, *p<0.05, **p<0.01). (**F**) Bar graph of mean values (mean ± SEM) of transcript fold change obtained by quantitative real-time PCR for *Bax*, *Bnip3*, *Gadd45*, *LC3*, and *Beclin-1* genes in different experimental groups at 3 dpi (n=4, ANOVA, post hoc Bonferroni *p<0.05).

In addition to SIRT1, we previously observed that NeuroHeal may modulate the PI3K/AKT pathway in some conditions in adult MNs 2. We wanted to analyze whether this pathway is involved in NH-induced neuroprotection in axotomized neonatal motoneurons as well. We found that neonatal axotomy provoked an increase of pAKT that was sustained by NeuroHeal treatment alone or in combination with NAM (Figure 3A; *p*<0.05). This result suggested that any effect on the PI3K/AKT pathway was independent of the SIRT1 activation induced by NeuroHeal. To confirm the activation of the PI3K/AKT axis, we analyzed further down. A main target of Akt is tuberous sclerosis 2 (TSC2) which forms heterodimeric complex with TSC1 and inhibits mTORC1, part of the mTOR complex. Phosphorylation of TSC2 by Akt blocks its activity and therefore allow activation of mTORC1which directly phosphorylate P70S6k (pP70S6k) 33. By immunohistochemical analysis we observed that the intensity of pP70S6k within MNs raised notably after injury compared to control (Figure 3B; *p*<0.01). The average intensity in axotomized MNs when animals were treated with NeuroHeal diminished respect to vehicle treated group (Figure 3B; *p*<0.01), what was abolished by co-treatment with NAM (Figure 3B; *p*<0.05). Altogether, these observations suggested that axotomy caused activation of PI3K/AKT/mTOR axis. Although PI3K/AKT activation is sustained by NeuroHeal treatment, downstream SIRT1 activation by this drug attenuates mTOR activation.

Considering a possible key point of convergence between AKT and SIRT1 in the action of NeuroHeal, we next analyzed FOXO3a, a member of the O subclass of the forkhead family of transcription factors, which is both an AKT substrate 34 and it can be deacetylated by SIRT1 35. It has been reported that FOXO3a may drive cell survival or death 36,37. In the neonatal *in vivo* model, injury caused an increase in the isoform of FOXO3a phosphorylated at Ser 253 relative to total FOXO3a (pFOXO3a/FOXO3a ratio), which was sustained by NeuroHeal treatment. The co-administration of NAM tended to normalize the relative levels of pFOXO3a although there were no differences with the treatment of NeuroHeal alone (Figure 3A;* p*<0.05). Despite immunoblotting analysis did not reveal quantitative differences, qualitative differences were observed by immunohistochemistry since the levels of FOXO3a within axotomized MN nuclei were drastically reduced by NeuroHeal treatment and they were restored by co-treatment with NAM (Figure 3C;* p*<0.01).

In contrast to what observed in pups, in SOCs, we observed an increase of the pFOXO3a/FOXO3a ratio upon NeuroHeal treatment respect to control and vehicle, that was abolished when cultures were co-treated with either NAM or EX-527, an specific SIRT1 inhibitor (Figure 3D;* p*<0.05).

Regarding the differences in localization observed in the pups and considering that not only phosphorylation but also acetylation influences the activity and nuclear localization of FOXO3a 36, we investigated the extent of acetylation by immunoblotting with anti-pFOXO3a after performing immunoprecipitation against anti-acetyl-lysine proteins. The level of acetylated pFOXO3a (AC-pFOXO3a) was significantly higher after injury with respect to control, but NeuroHeal treatment normalized its levels *in vivo* (Figure 3E;* p*<0.05). Similarly, in SOCs NeuroHeal treatment drastically reduces the levels of AC-pFOXO3a (Figure 3E;* p*<0.01).

Since the presence or absence of FOXO3a within the nucleus may alter downstream gene expression, we subsequently analyzed the expression of several genes known to be regulated by this transcription factor such as *Bax* 38, *Bnip3* 39, *Gadd45* 40, *Lc3*, and *Beclin1* 41 in the *in vivo* model*.* Neonatal axotomy increased levels of almost all of them except *Beclin1* for which the trend did not achieve statistical significance (Figure 3F;* p*<0.05). NeuroHeal treatment significantly reduced levels of *Bax* and *Bnip3*, genes related to apoptosis, and this reduction was blocked by NAM co-treatment. Sustained expression of *Gadd45* by NeuroHeal treatment was also blocked by NAM co-administration. In contrast, NAM did not alter the NeuroHeal treatment-induced up-regulation of pro-autophagy genes *LC3* and *Beclin1*.

Altogether, these results suggested that the concurrent activation of SIRT1 and AKT pathways when treatment with NeuroHeal induces nuclear localization of acetylated pFOXO3a, which selectively reduces the expression of some FOXO3a-downstream pro-apoptotic genes.

### Concomitant deacetylation and phosphorylation of FOXO3a is key to neuroprotection

**Figure 4:**
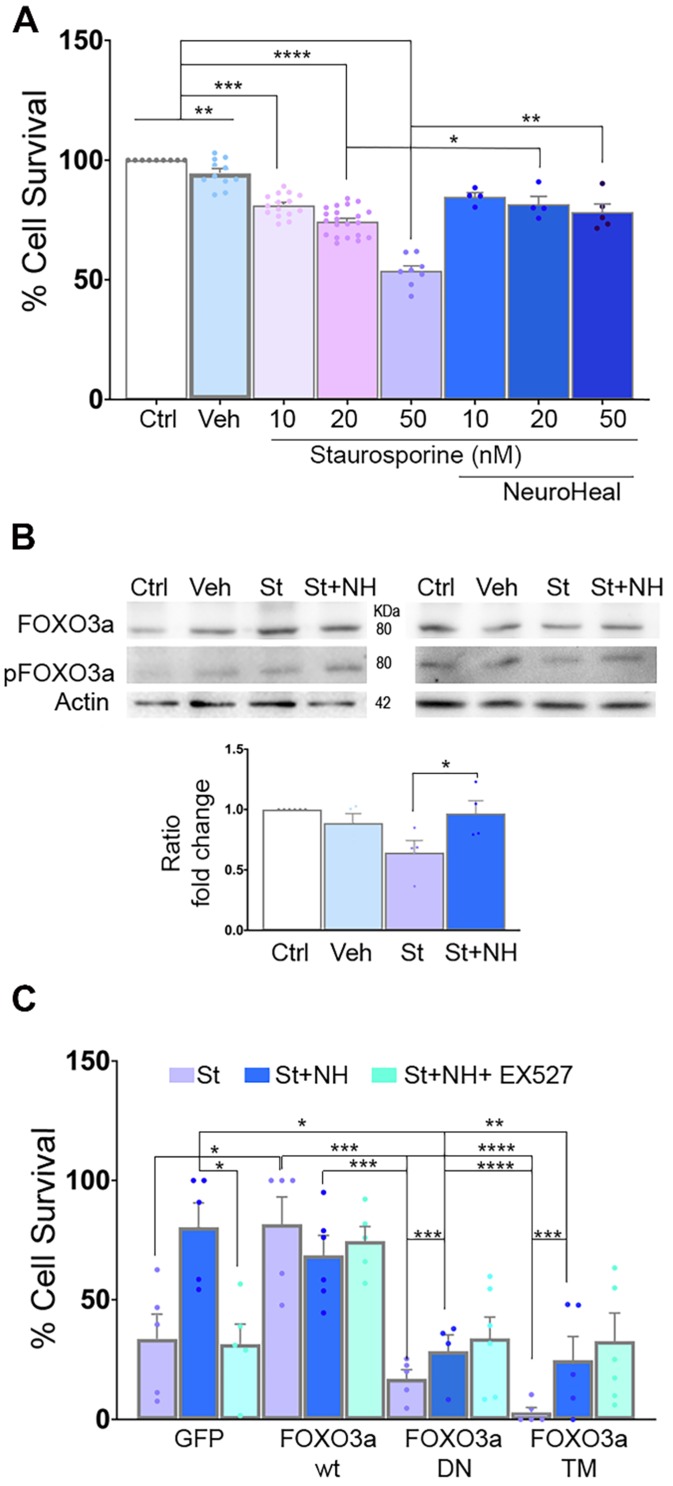
** FOXO3a mediates the neuroprotective effect of NeuroHeal.** (**A**) Bar graph showing the percentage of NSC-34 cell survival (mean ± SEM) after treatment with 10, 20, or 50 nM staurosporin (St) and with or without NeuroHeal (NH) (n=6-10, ANOVA, post hoc Bonferroni *p<0.05). (**B**) Western blots and bar graph showing the analysis of pFOXO3a respect to total FOXO3a in NSC-34 cell cultures (n=4 per group, ANOVA, post hoc Bonferroni, *p<0.0). (**C**) Bar graph showing the percentage of NSC-34 cell survival (mean ± SEM) relative to vehicle-treated cells after treatment with 50 nM St with or without NH (n=4 per group, ANOVA, post hoc Bonferroni *p<0.05*,* **p<0.01, ***p<0.005, ****p<0.001 ).

In order to establish the role of deacetylated pFOXO3a in the neuroprotective effect mediated by NeuroHeal against apoptosis, we used an *in vitro* model based on the use of staurosporine (St) as a pro-apoptotic agent 42. We confirmed that St decreased survival of NSC-34 MN-like cells in a dose-responsive manner and we found that NeuroHeal was neuroprotective at the highest concentration of St (Figure 4A; *p*<0.01).

Immunoblotting analysis showed an increase pFOXO3a/FOXO3a ratio by NeuroHeal co-treatment respect to St treatment alone (Figure 4B; *p*<0.05).

To further establish the importance of pFOXO3a in survival of apoptotic neurons, we transfected NSC- 34 cells with: a vector for overexpression of either the wild-type (WT) FOXO3a, a dominant negative mutant (FOXO3a-DN), or a triple mutant form (FOXO3a-TM) (Figure 4C). FOXO3a-DN, constructed by deletion of the transactivation domain from the C-terminus, competes with wild-type FOXO3a for binding to DNA 43. The FOXO3a-TM yield a non-phosphorylatable (with alanine residues at positions Thr32, Ser253 and Ser315) and, hence, constitutively inactive form of FOXO3a (TM-FOXO3a) 43. We verified the expected effect of mutants analyzing FOXO3a protein size and subcellular location by immunoblot. As shown in Figure S2A, FOXO3a was detected in both cytoplasmic and nuclear fractions in transfected cells with either WT or GFP plasmids at the expected size (around 80 KDa). In FOXO3a-DN cells, which yield a deleted product, the band was smaller size as expected in both fractions 43. We also verified reduced levels of BIM protein, depending on FOXO3a activity, in DN and drastically, in TM mutants. Transfection of the cells with these vectors did not affect survival when compared to GFP group (Figure S2B). Overexpression of wild-type FOXO3a promoted survival itself after St administration similar to NeuroHeal treatment, whereas survival was drastically reduced in cells overexpressing FOXO3a-DN or FOXO3a-TM in all treatment conditions (Figure 4C;* p*<0.001). These results suggested that the levels of phosphorylated form of FOXO3a and its activity are important for neuroprotection mediated by NeuroHeal in the staurosporine-induced apoptosis paradigm.

### SIRT1 and AKT activations are necessary for neuroprotective autophagy

**Figure 5:**
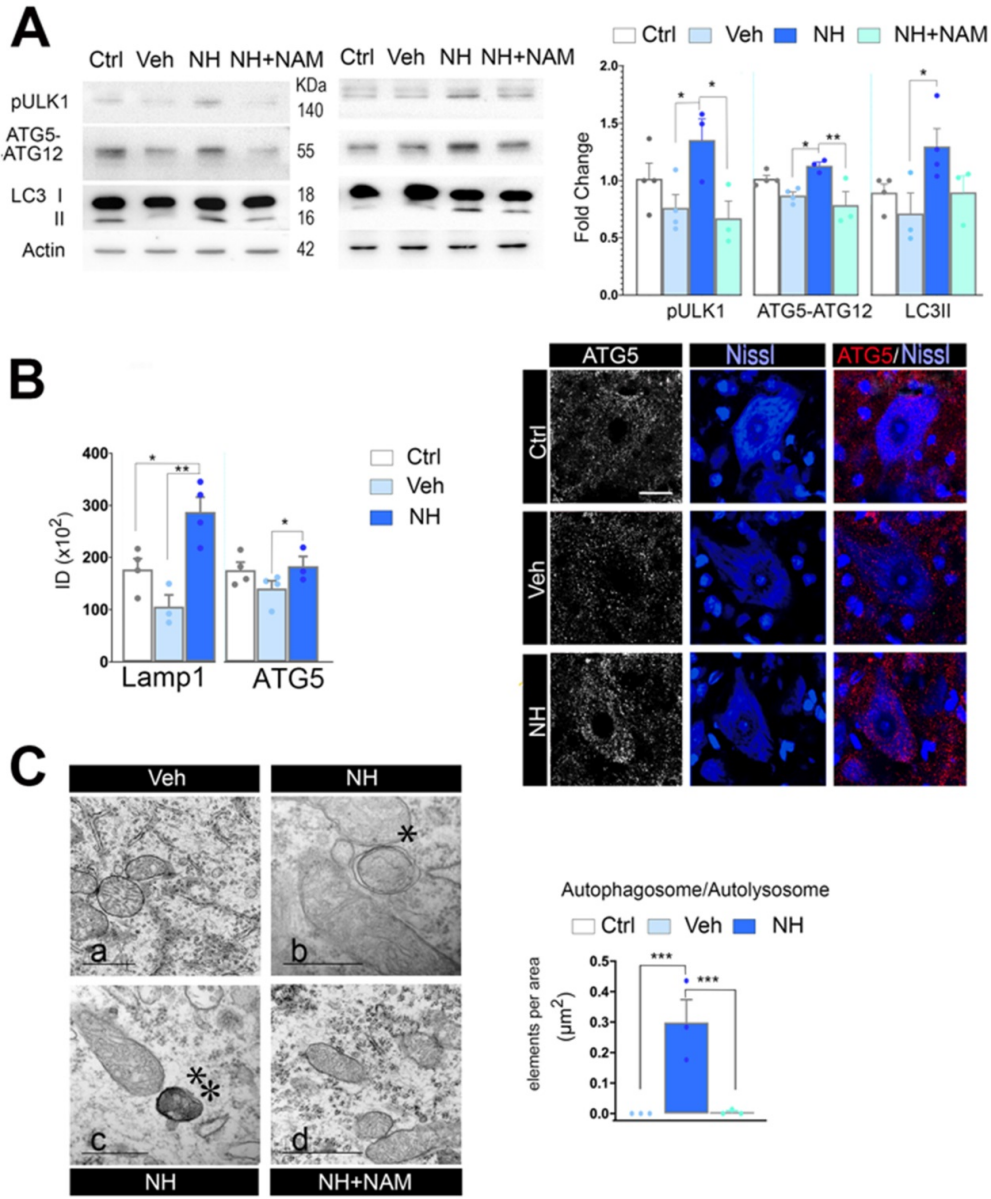
** NeuroHeal activates SIRT1-dependent autophagy. (A)** Western blots and bar graphs showing the analysis of pUlk1, ATG5-ATG12, and LC3II levels in different experimental groups at 3 dpi (n=4 per group, ANOVA, post hoc Bonferroni, *p<0.05, **p<0.01). **(B) *Left,***Bar graphs of the mean immunofluorescence intensity for each marker within the MNs (n=4 animals per group, *t-test*, *p<0.05, **p<0.01). ***Right,***Representative confocal microphotographs of samples ATG5 (red), and counterstained with FluoroNissl (Blue) from contralateral side (Ctrl) and injured side of vehicle-treated (Veh) and NH-treated animals. Scale bar 25 µm. (**C**) ***Left****,* Representative transmission electronic images within MNs form the different animal groups at 3 dpi. Note that only autophagosomes* or autolysosomes**, are detected in NH-treated MNs. Scale bar: a= 1 µM, b & c= 500 µM, d= 1000 µM.*** Right,***Bar graph of the number of autophagosomes and autolysosomes found within MNs at each group per area (*t-test*, ***p<0.005).

The apoptosis/autophagy balance may be determinant of cell fate and several factors are known to influence the autophagy/apoptosis balance 44. Since FOXO3a favored the expression of autophagy- related genes, we analyzed for activation of autophagy after neonatal injury. In cells undergoing autophagy, phagophore formation initiates after the Unc-51 like autophagy activating kinase 1 (ULK1) activation, and its elongation is regulated by two ubiquitin-like reactions: the first leading to the formation of the complex ATG12-ATG5-ATG16L1; and the second, involves the conjugation of the microtubule-associated protein light chain 3 (MAP- LC3/ATG8/LC3) to phosphatidylethanolamine at the autophagosome membrane to form autophagosome- associated LC3-II. Once, the autophagosome is formed, it acquires the ability to bind autophagic substrates and/or proteins that mediate cargo selectivity (including sequestosome 1 (p62/ SQSTM1)). Autophagosomes mobilize toward lysosomes along microtubules; then, the outer membrane of the autophagosome fuses with the lysosome, thanks to lysosomal proteins like LAMP1, to form an autolysosome 45. We evaluated autophagy markers Ulk1 phosphorylated at Ser 555, conjugated ATG5-ATG12, and LC3II, as previously described by immunoblotting spinal cord extracts at 3 dpi and perform quantitative western analysis. Neonatal axotomy did not affect the levels of these proteins, whereas there were a significant increase in all of them in the NeuroHeal treated group compared to vehicle. The addition of NAM abolished all these effects observed in the NeuroHeal group (Figure 5A;* p*<0.01). Accordingly, by immunohistochemical analysis we observed abundant dot-like structures which were positive for LAMP1 within the cytosol of axotomized MNs preferently in the NeuroHeal group and the overall immunofluorescence intensity was higher in this group than in the vehicle and control ones (Figure S3 & 5B). Although the increase in LAMP1 might be related also to lysosomal biogenesis, we observed similar increase and dot-like pattern for ATG5 staining in NeuroHeal-treated animals (Figure 5B). Correspondingly, by electron microscopy we confirmed the presence of autophagosomes and autolysosomes in MNs only from injured animals treated with NeuroHeal (Figure 5C;* p*<0.005).

**Figure 6:**
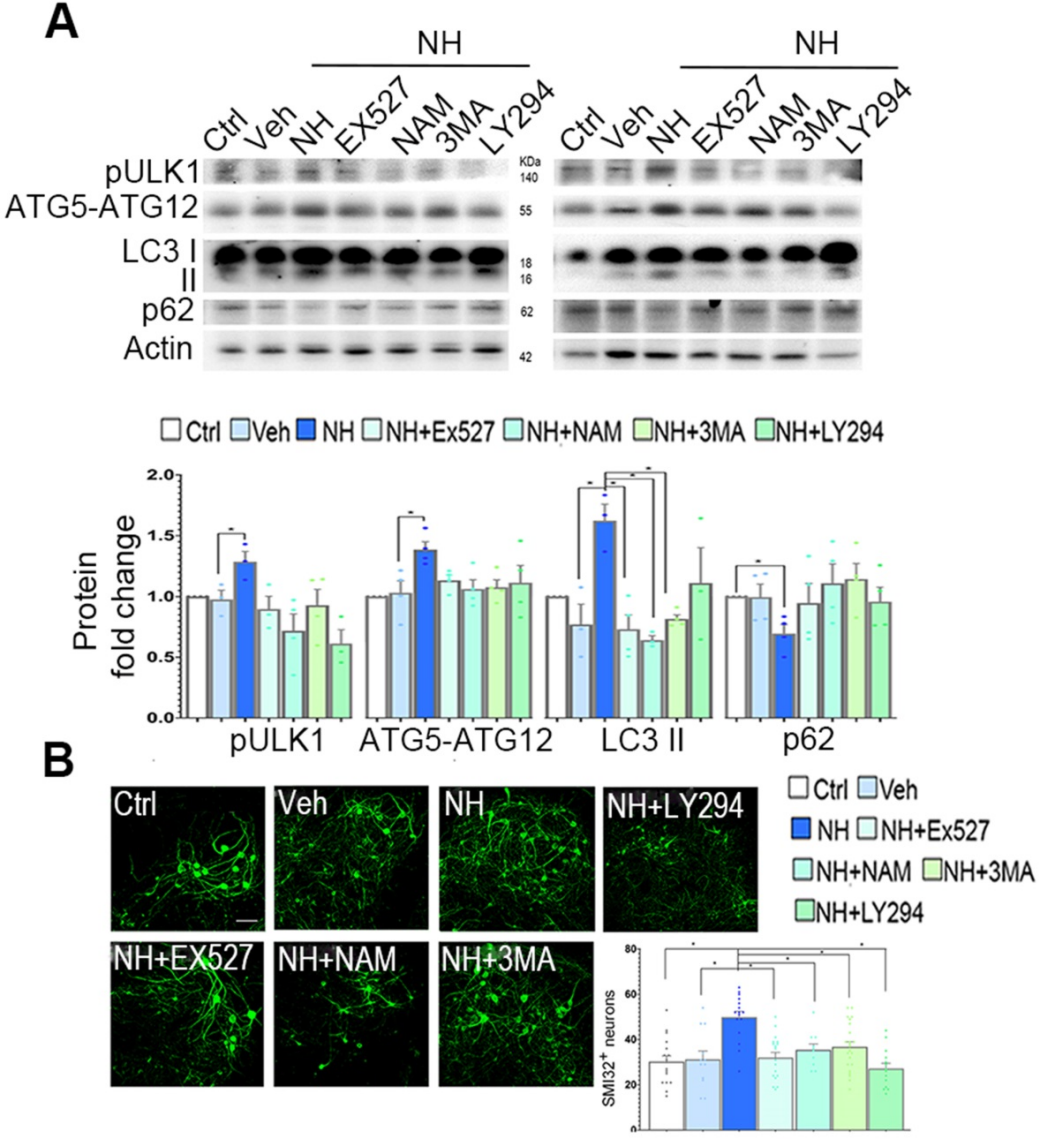
** Neuroprotective autophagy triggered by NeuroHeal depends on co-activation of AKT/SIRT1 axes. (A)** Western blots and bar graph showing the analysis of pUlk1, ATG5-ATG12, and LC3II protein levels from differentiated SOCs after 2 days of treatment with vehicle or NeuroHeal (NH) alone or in combination with SIRT1 or PI3K inhibitors (3MA and LY294) (n=4 per group, ANOVA, post hoc Bonferroni, *p<0.05). **(B)** Representative microphotographs of SMI32 immunolabeled differentiated motoneurons at the ventral horn of the SOCs treated for 2 days at the different conditions. Scale bar 100 µm. Bar graphs showing the numbers (± SEM) of SMI-32-positive cells (green) in the ventral horns of each hemi-section of the spinal cord slice at the different conditions (n=8-12 complete spinal cord slices, ANOVA, post hoc Bonferroni *p<0.05). Scale bar 100 µm.

In SOCs, we observed similar effects of NeuroHeal treatment on LC3II, pUlk1, and ATG5-ATG12 levels (Figure 6A). The increases due to NeuroHeal treatment were abolished when using the inhibitors of phosphatidylinositol 3 kinases such as 3-methyladenine (3MA) or LY294, or with SIRT1 inhibitors EX-527 or NAM (Figure 6A). Lastly, we observed a significant reduction in the levels of p62/SQSTM1 (selectively degraded by autophagy) suggesting absence of autophagic flux blockage. Both SIRT1 inhibitors increased the acetylated forms of p53 and H3 as expected (Figure S4;* p*<0.05). Moreover, the NeuroHeal-induced neuroprotection was abolished when cultures were co-treated with any of these inhibitors (Figure 6B;* p*<0.05). We also verified the importance of autophagy-mediated induction by Neuroheal in neuroprotection against apoptosis by treating NSC34 cells with bafilomycin (Baf). Baf is potent (V)-ATPase inhibitor that interrupt the autophagosome-lysosome fusion step autophagy. As sown in Figure S5, only cells treated with NeuroHeal had higher cell viability than those treated with St, what was abolished when Baf was added too. These observations corroborate the importance of autophagy induction by NeuroHeal for neuroprotection.

These results indicate that both SIRT1 and PI3K/ AKT activation are required by NeuroHeal-mediated neuroprotection and the induced autophagy- dependent process.

## Discussion

Obstetric complications like brachial plexus avulsion during birth lead to axotomy of MNs and consequent mobility problems for life. Nerve surgical repair may palliate the consequences only if performed early enough to avoid the rapid decline of disconnected MNs. The immature neuronal system does not behave identically at the molecular level as the mature system. Because of that, neuroprotective agents well performing in the adult should prove to be effective also during development if advisable for pediatric use. We aimed to test whether NeuroHeal treatment may exert neuroprotection to axotomized neonatal MNs that succumb by apoptosis, mainly dependent on Casp 3, and elucidate the mechanism of action involved. We discovered that NeuroHeal induces fine-tuned autophagy and concomitant activation of SIRT1 and AKT/PI3K axes converging onto FOXO3a, as a necessary node for neuroprotection.

We previously found that NeuroHeal is a neuroprotective agent for disconnected MNs in different models of nerve injury in adult rodents 2,46. Adult spinal MNs that suffered from root avulsion or proximal axotomy suffer a slow neurodegenerative process marked by a non-canonical UPR response and defective autophagy flux 6,8. In this context, we observed that NeuroHeal activates SIRT1, and increased integrins and cytoskeletal and motor-related proteins such as dynactin and Kinesin 5c 2.Herein, we observed that NeuroHeal exerted also neuroprotection to apoptotic MNs. The mechanism involved may interfere with the final substrates of Casp3 since persistent activation of this caspase was observed event after NeuroHeal treatment despite neuroprotection. So, in which manner can NeuroHeal arrest apoptosis or drive the cell towards other pro-survival signals?

We observed that Neuroheal fine-tunes autophagy induction which was necessary to exert its neuroprotection and it is conducted by opposite forces. NeuroHeal activated SIRT1 and SIRT1- dependent autophagy 47-49. On the other hand, NeuroHeal sustained AKT activation after injury which normally inhibits autophagy primarily by activating mTOR 20. Surprisingly, NeuroHeal treatment attenuated mTOR activation in axotomized neonatal MNs what would indeed favor the autophagy induction that we observed. How might this be? Several reports have described that SIRT1 activation may interfere with PARP1 and mTOR activities that altogether seem to form a tripartite key node to orchestrate cell destiny 50. SIRT1 deacetylates PARP1 blocking its catalytic activity 51,52. SIRT1 blocks mTOR activity indirectly, probably by deacetylation and activation of TSC2, an inhibitor of mTOR 53. It may happen that activation of SIRT1 by NeuroHeal blocks both PARP1/2 and mTOR, and consequently facilitating cell survival, since overactivation of PARP1/2 can lead to cell death 54, and pro-survival autophagy 33, respectively. Besides, PARP1/2 can be degraded by autophagy 32. However, further experiments should be performed to confirm all these details that may be involved. Nevertheless, we have validate previous in silico prediction about NeuroHeal claiming PARP1/2 as one key node possible target mechanism of action 2.

Both factors, SIRT1 and AKT, interact with FOXO3a, a transcription factor whose regulation depends on multiple post-translational modifications that determine both the subcellular localization and activity 55. We established herein that concomitant phosphorylation and deacetylation of FOXO3a in axotomized neurons drive a “FOXO code” that downregulates apoptotic genes while preserving the expression of autophagy-related ones. Our results are in agreement with a study in hematopoietic stem cells that indicated that FOXO3a regulates a genetic program that results in activation of autophagy under stress conditions to allow survival 56. Our data also agree with reports that FOXO3a deacetylation by SIRT1 leads to the expression of particular genes, like *Gadd45* 57, but not others, such as *Bim* 37. We also established that the phospho-form of FOXO was necessary for the neuroprotection established by NeuroHeal against apoptosis.

Finally, a question that remains is how autophagy induction may exert neuroprotection in this model. Whether autophagy is a cell death or a pro-survival mechanism is still controversial. Some authors working in the field of hypoxia-ischemia (HI) in neonates have observed that autophagy is strongly induced after damage, and consequently, blocking it yield neuroprotection 58,59. Although some claimed that autophagy pathways are disturbed in regional- and sex-specific patterns in the rat brain following neonatal HI reinforcing the idea that many factors influence 60. Indeed, it appears that autophagy apparition and modulation by any agent would depend on the brain cell type and/or nutrient availability, i.e., the activation status of autophagy (basal vs. induced). Besides, neonatal HI triggers both caspase-dependent and independent cell-death 61 and hence may be different than what accounts to axotomized neonatal MNs. Peripheral axotomy in neonates induces excitotoxicity and oxidative stress leading to DNA damage, mitochondria function fail and energy depletion on damaged MNs. Although it is speculative, the induction of mild autophagy as with NeuroHeal may proportionate energy from enhancing the clearance of dysfunctional proteins and organelles like mitochondria or diminish excitotoxicity-induced DNA damage, as it has been described in several models of neurodegenerative diseases 62.

A recent study, it has been observed that excitotoxicity blocks autophagy triggered in cells previously to the insult by starvation. These authors concluded that appears that autophagy modulation by gluta- mate might be more complex then previously anticipated, including both stimulatory and inhibitory effects depending on the brain cell type and/or nutrient availability, i.e. the activation status of autophagy (basal vs. induced). We can add to this the condition of immaturity of neonatal neurons were the channels receptors and signaling machinery are not exactly as the mature neuron.

## Conclusions

We conclude that NeuroHeal is a neuroprotective agent that sustains the activation of both SIRT1 and PI3K/AKT converging to promote a specific coded FOXO3a function to avoid neuronal demise. In light to overall results, we propose NeuroHeal as a neuroprotective agent for pediatric use.

## Supplementary Material

Supplementary figures and tables.

## Abbreviations

AC-pFOXO3a: acetylated pFOXO3a
DIV: days of *in vitro* culture
dpi: days post injury
FOXO3a-DN: dominant negative mutant
FOXO3a-TM: triple mutant form
H3K9Ac: histone H3 acetylated at Lys9
MN: motoneuron
MTT: 3-(4,5-dimethylthiazol-2-yl)-2, 5-diphenyltetrazolium bromide
NAM: nicotinamide
PI3K: phosphatidylinositol-4,5-bisphosphate 3-kinase
p53Ac: p53 acetylated at Lys373
SIRT1: sirtuin 1
SOC: spinal organotypic culture
St: staurosporine
TBS: Tris-buffered saline
TSC2: tuberous sclerosis 2
UPR: unfolded protein response
