# Supplementary material for: Novel neuroprotective therapy with NeuroHeal by autophagy induction for damaged neonatal motoneurons
Source: Theranostics. 2020 Apr 6;10(11):5154–68. doi: 10.7150/thno.43765 (PMC7163445; doi:10.7150/thno.43765)

Novel Neuroprotective Therapy with NeuroHeal by Autophagy Induction for Damaged Neonatal Motoneurons

David Romeo-Guitart, César Marcos-DeJuana, Sara Marmolejo-Martínez-Artesero, Xavier Navarro, and Caty Casas*

Affiliations

Institut de Neurociències (INc) and Department of Cell Biology, Physiology and Immunology, *Universitat Autònoma de Barcelona* (UAB), & *Centro de Investigación Biomédica en Red sobre Enfermedades Neurodegenerativas* (CIBERNED), Bellaterra, Barcelona, Spain.

*To whom correspondence should be addressed: Caty Casas Louzao, *Unitat de Fisiologia Mèdica*, *Facultat de Medicina*, *Universitat Autònoma de Barcelona*, E-08193 Bellaterra, Barcelona, Spain. Tel: +34-935811324, Fax: +34-935812986, E-mail: Caty.Casas@uab.cat

SUPPLEMENTARY INFORMATION

- **Supplementary Tables**

Table S1. Primary antibodies for immunohistochemistry

Antibody name	Reference
rabbit anti-Iba1	1:1000, Wako, ref 1919741
rabbit anti-SIRT1	1:100, Millipore, ref 07-131
rabbit anti-H3K9Ac	1:50, Millipore, ref 06-942
rabbit anti-p53-K373Ac	1:500, Millipore, ref 06-916
rabbit anti-cleaved Casp3	1:200; Cell Signaling, ref 96615
rabbit anti-LAMP1	1:200, Antibodies Online, ref 3187789
mouse anti-ATG5	1:200, Nanotool, ref 0263-100
mouse anti-p62	1:100; BD Transduction Laboratories, ref 610833
rabbit anti-phospho-FOXO3a (Ser253)	pFOXO3a; 1:500, Abcam, ref ab 31109-100
rabbit anti-FOXO3a	Novus Biologicals, 1:200, ref NBP2-16521
rabbit anti-phospho-S6 kinase (Thr389)	pP70S6k; 1:1000, Antibodies Online, ref 863186
rabbit anti-PARP1/2	1:200; Santa Cruz, ref sc-7150
anti-β-III-tubulin	1:500, Covance, ref MMS-435P/801202
anti SMI32	1:1000; Biolegend, ref 801701

Table S2. Primary antibodies for Western Blot

Antibody name	Reference
rabbit anti-PARP1/2	1:500; Santa Cruz Biotechnology, ref sc-7150
rabbit anti-phospho-Ulk1 (Ser 555)	pUlk1; 1:1000; Millipore, ref ABC124
mouse anti-Atg5	1:1000; Nanotools, ref 0263-100
rabbit anti-LC3	1:1000; Abcam, ref ab48394
rabbit anti-pFOXO3a	1:1000, Abcam, ref ab31109-100
rabbit anti-FOXO3a	Novus Biologicals, 1:500, ref NBP2-16521
rabbit anti-phospho-AKT	pAKT; 1:1000; Cell Signaling, ref 4060

rabbit anti-AKT	1:1000; Cell Signaling, ref 4691
Mouse anti-β-actin	1:3000, Sigma Aldrich, ref A5316

Table S3. Primers used for RT-PCR

Primer Name	Sequence (5' to 3')
rBNIP3-F	CAGAGCGGCGAGGAGAACCTGCAG (24)
rBNIP3-R	GCTGCTCCCATTCCCATTGCTGAAG (25)
rBeclin-F	GGTAGCTTTTCTGGACTGTGTGCAGCAG (28)
rBeclin-R	GTCTTCAATCTTGCCTTTCTCCACGTCC (28)
rLC3-F	ACCCTCCCTGCATGCAGCTGTCC (23)
rLC3-R	ACCAGGGACATGACGACGTACACAACC (27)
rGADD45-R	TGAGGGTGAAATGGATCTG (19)
rGADD45-F	GAAGATCGAAAGGATGGAC (19)
rBax2-F	CTGCAGAGGATGATTGCTGA (20)
rBax2-R	GATCAGCTCGGGCACTTTAG (20)
rActin-F	TCAGGTCATCACTATCGG (18)
rActin-R	CAGTAATCTCCTTCTGCATC (20)

- **Supplementary Figures and Legends**

Supplementary Figure S1

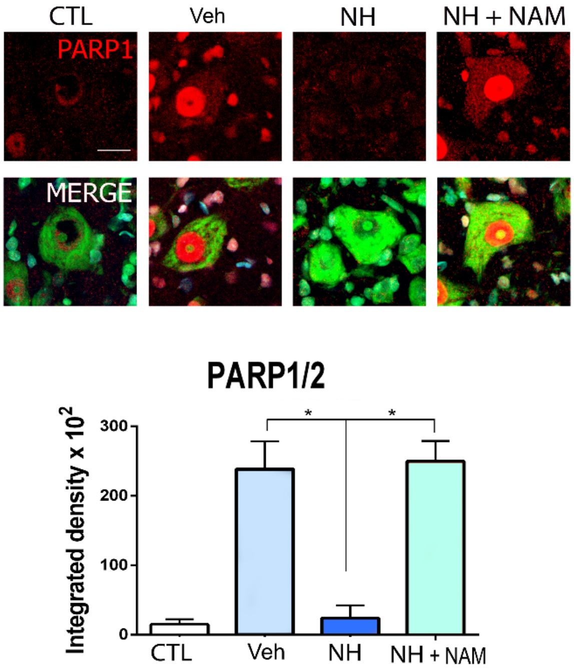

Supplementary Figure S1. ***Top,*** Representative confocal microphotographs of the immunolabelling of PARP1/2 (red), and FluoroNissl (green), and DAPI (blue) in merged pictures of MNs from different experimental conditions at 10 dpi. Scale bar 20 µm. ***Bottom,*** Quantification of the integrated density of PARP1/2 in nuclei of MNs (n=4 per group, ANOVA, post hoc Bonferroni, *p<0.05).

Supplementary Figure S2

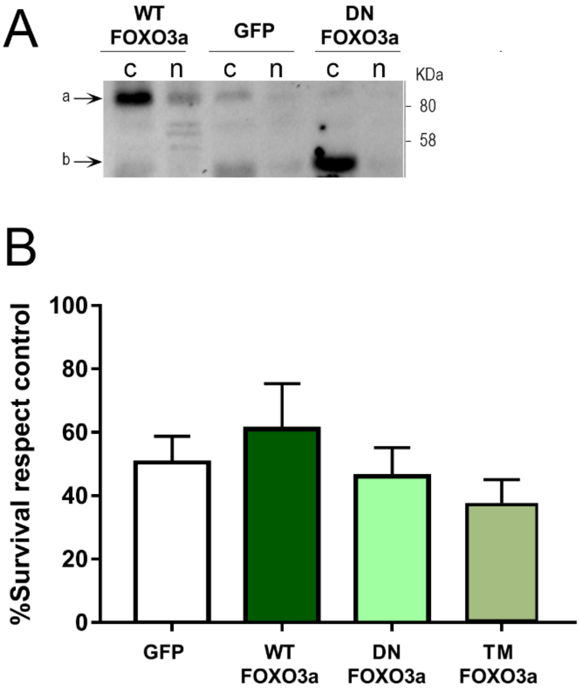

Supplementary Figure S2. (A) Representative images from two independent experiments of immunoblots to detect FOXO3a (top) or BIM (down) from NSC34 cells either untransfected (CTL) or nucleofected with either GFP, double negative (DN) or nonphosphorylatable triple mutant (TM) plasmids. Note that FOXO3a corresponding band is normally detected above 80 Kda (band a) except when expression of the delected form of DN mutant (<50KDa, band b). (**B**) Graph showing the percentage survival (± SEM) of transfected NSC-34 cells with respect to cells transfected with control vector analysed at 4 DIV.

Supplementary Figure S3

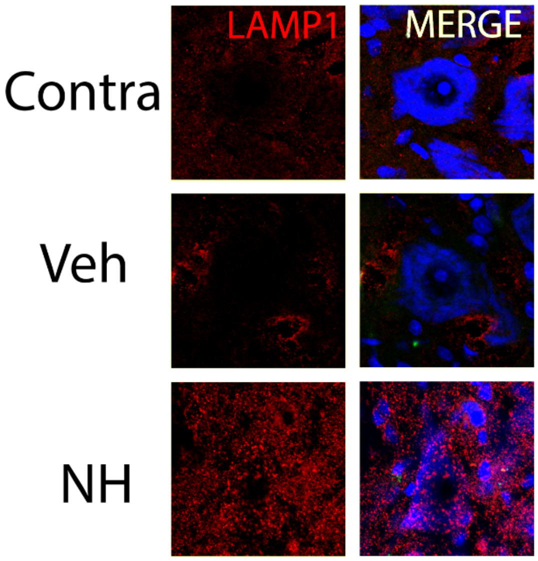

Supplementary Figure S3. Confocal microphotographs of samples stained for LAMP1 (red), and counterstained with FluoroNissl (blue) from a representative contralateral side and injured side of vehicle-treated and NH-treated injured animals at 10 dpi. Scale bar 25 μm.

Supplementary Figure S4

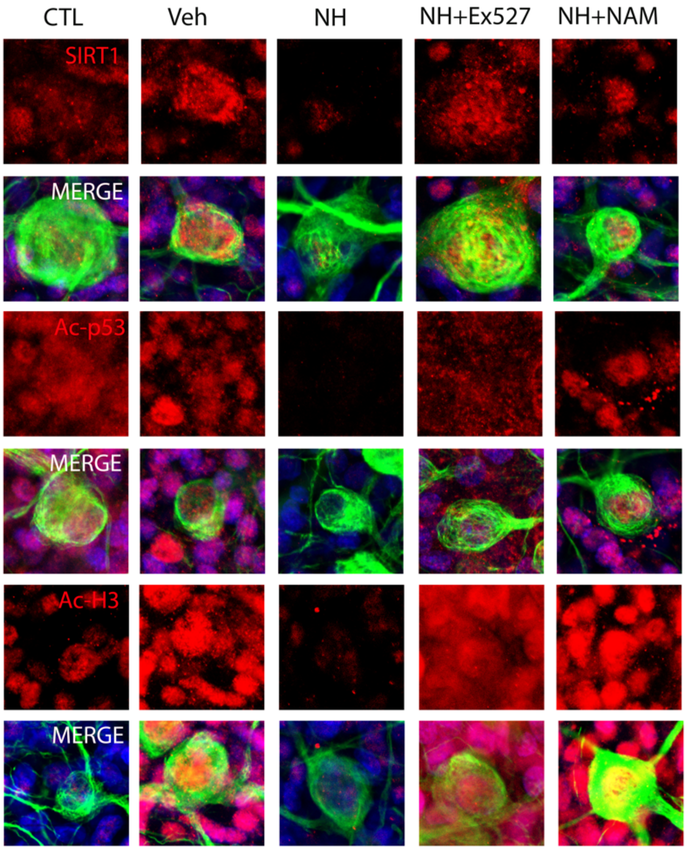

Supplementary Figure S4. Confocal microphotographs of SOCs immunolabelled for SIRT1, p53Ac, or H3K9Ac (red), SMI3-32 (green), and DAPI (blue) at 15 DIV after the treatment with vehicle, NeuroHeal (NH), or NH+SIRT1 inhibitors. Scale bar 25 µm.

Supplementary Figure S5

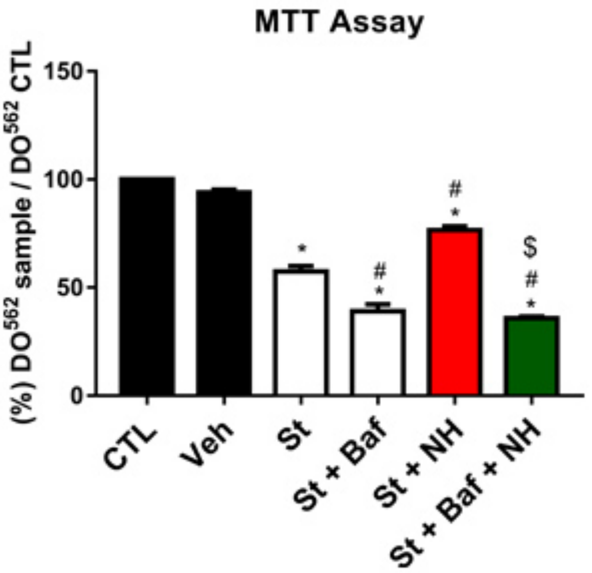

Supplementary Figure S5. Analysis of cell survival using MTT assay in different conditions: 100 µM Bafilomycin A1 (a V-ATPase inhibitor, Baf) to apoptotic cells induced by staurosporine (St) and treated with NeuroHeal (NH). Ctrl, control; veh1, 0.0002% DMSO; veh2, 0.01 % etOH.*p<0.05 *vs* Control;# p<0.05 *vs* St; $ p<0.05 *vs* St+NH.

- ## Supplementary Material

Extra Immunoblot Membranes for Fig 2B_ Casp3

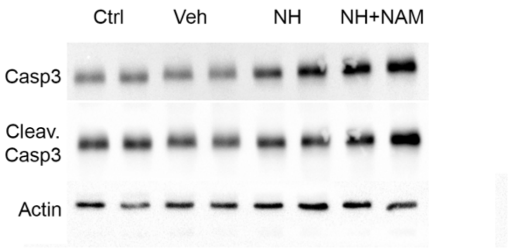

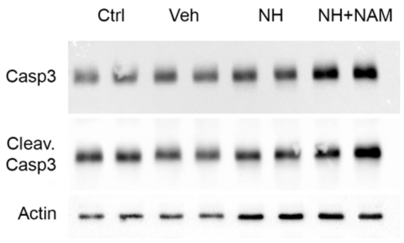

Supplement: Supplementary file 1 — Supplementary figures and tables. [file thnov10p5154s1.pdf]
